# Supplementary material for: Rapid and Non-Invasive Assessment of Texture Profile Analysis of Common Carp (Cyprinus carpio L.) Using Hyperspectral Imaging and Machine Learning
Source: Foods. 2023 Aug 22;12(17):3154. doi: 10.3390/foods12173154 (PMC10486347; doi:10.3390/foods12173154)

Figure S1. PCA plots clustering common carp with eight textural indicators of dorsal muscles.

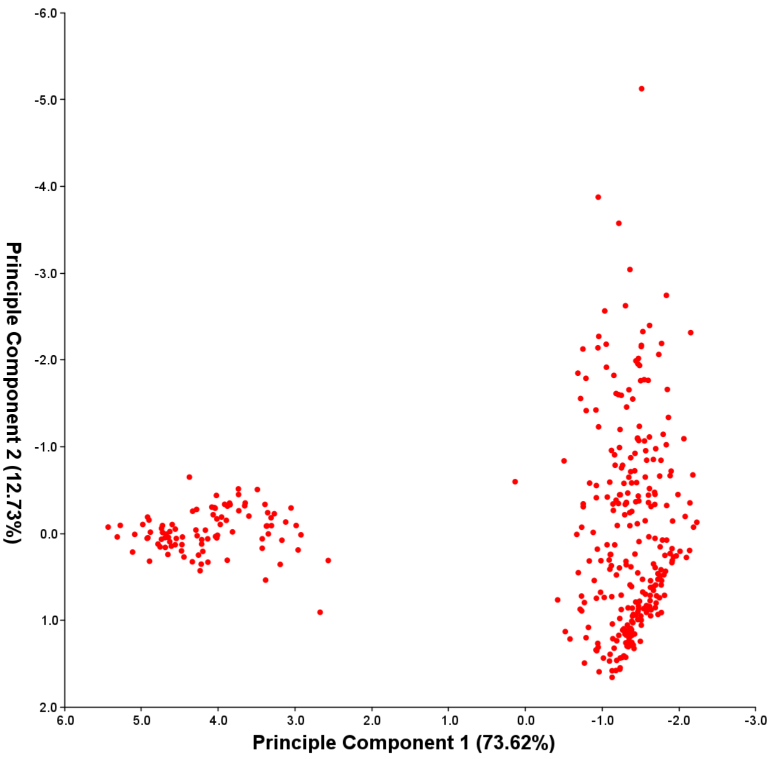

Figure S2. PCA plots clustering common carp with eight textural indicators of pectoral muscles.

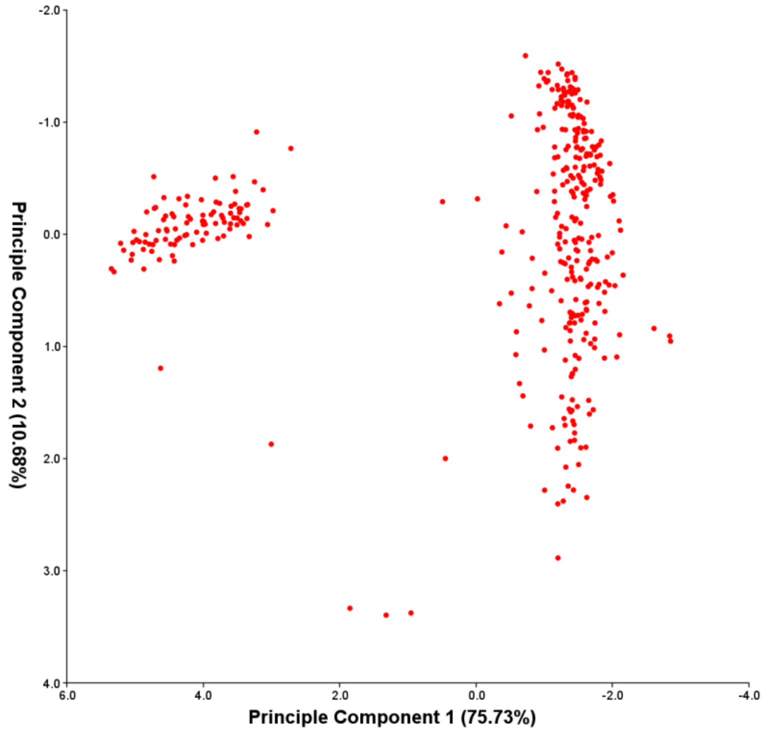

Figure S3. PCA plots clustering common carp with eight textural indicators of abdominal

muscles.

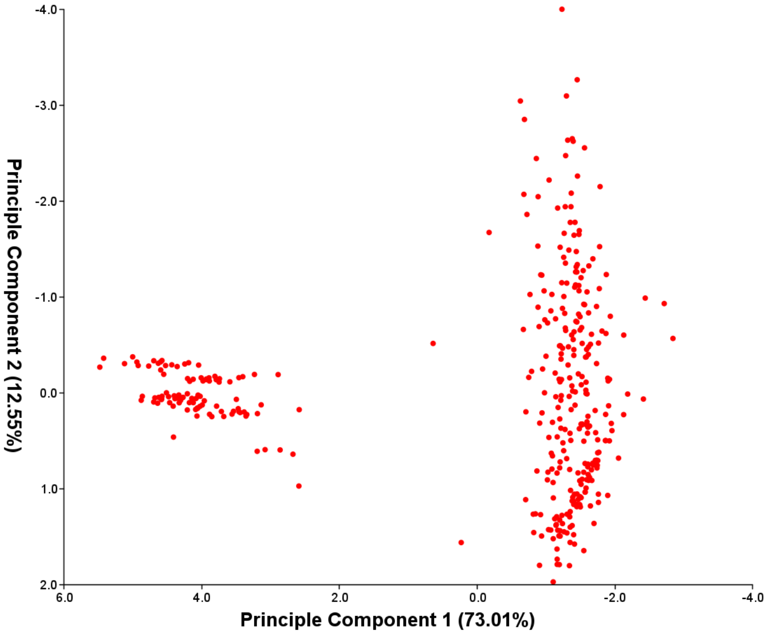

Figure S4. PCA plots clustering common carp with eight textural indicators of gluteal muscles.

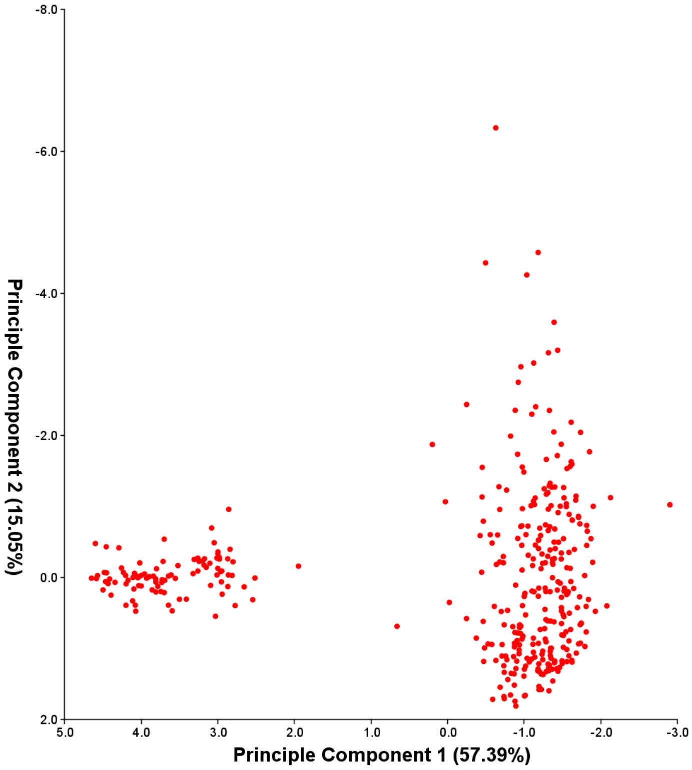

Figure S5. Box-plot of cohesiveness in the four muscle regions of common carp.

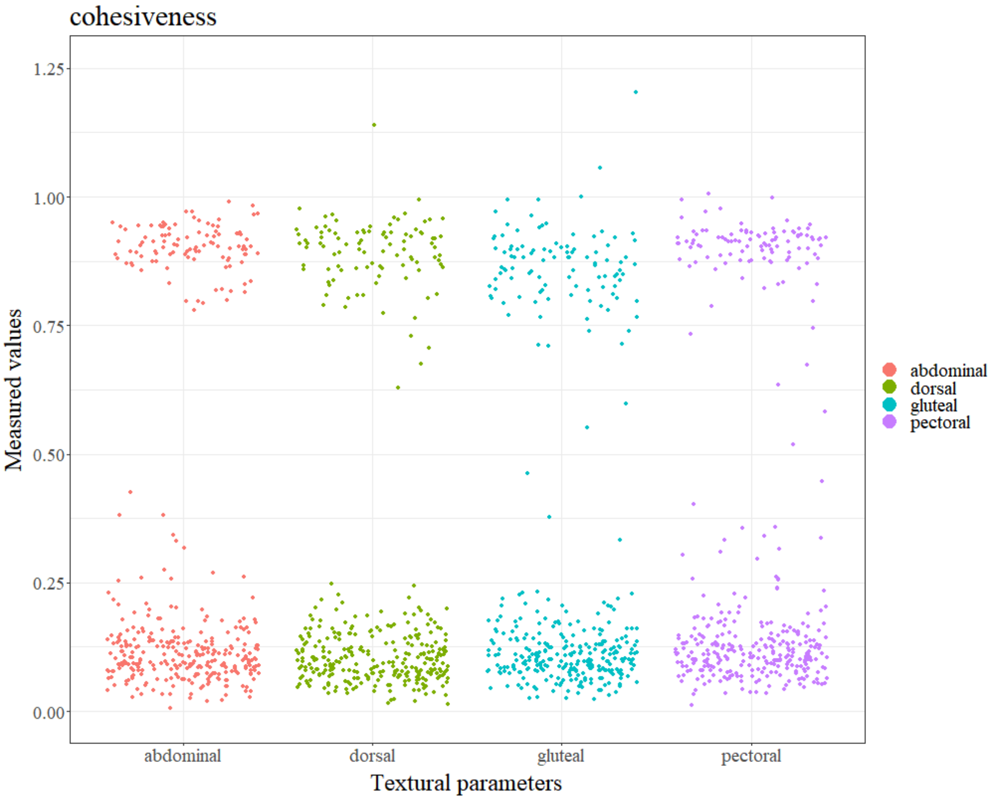

Figure S6. Scatter plot of adhesiveness values in the four muscle regions of common carp.

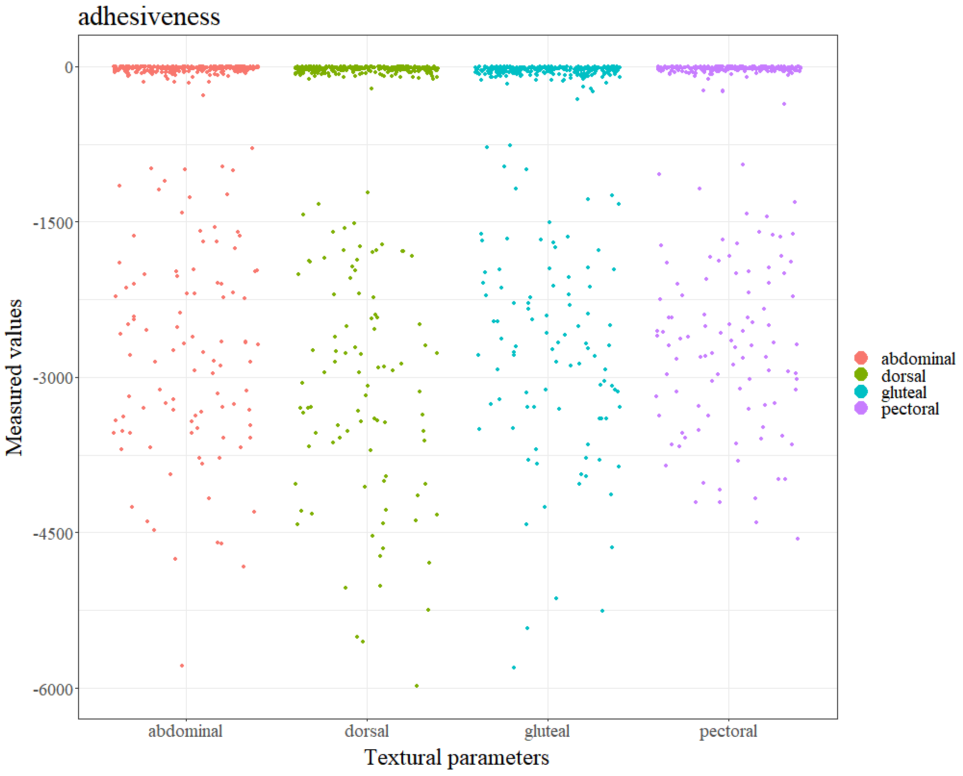

Figure S7. Scatter plot of chewiness values in the four muscle regions of common carp.

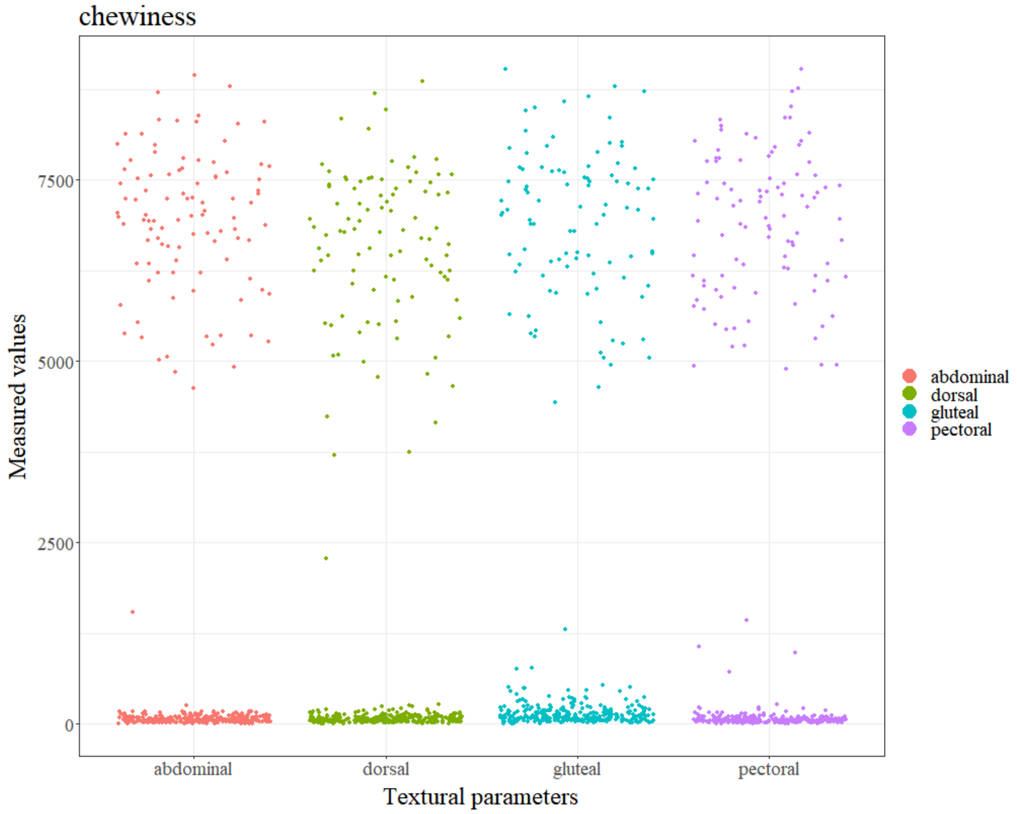

Figure S8. Scatter plot of gumminess values in the four muscle regions of common carp.

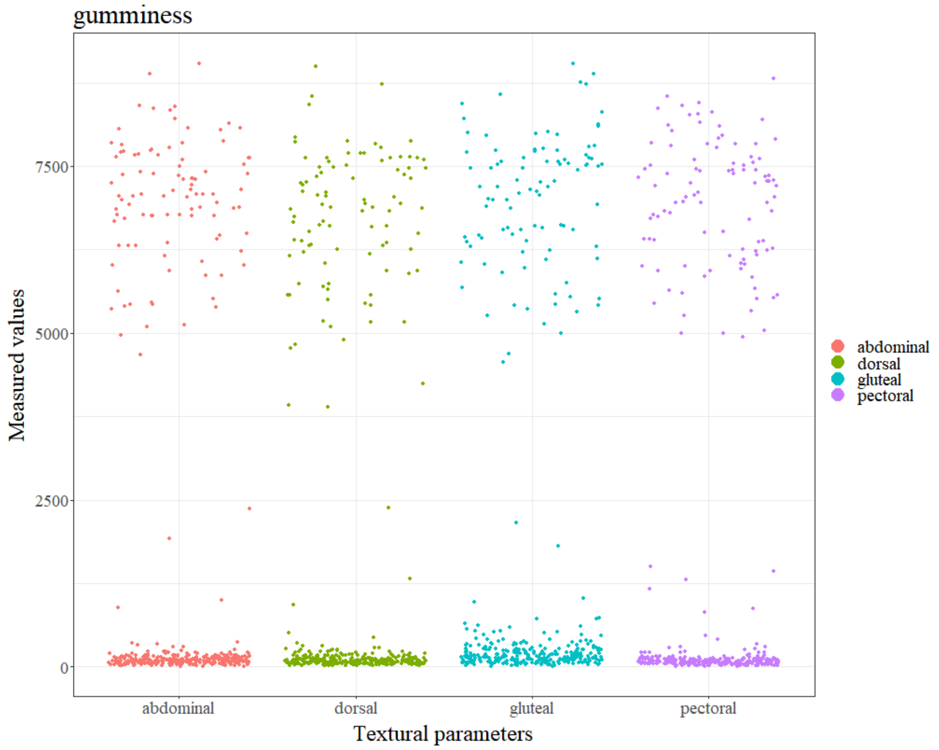

Figure S9. Scatter plot of resilience values in the four muscle regions of common carp.

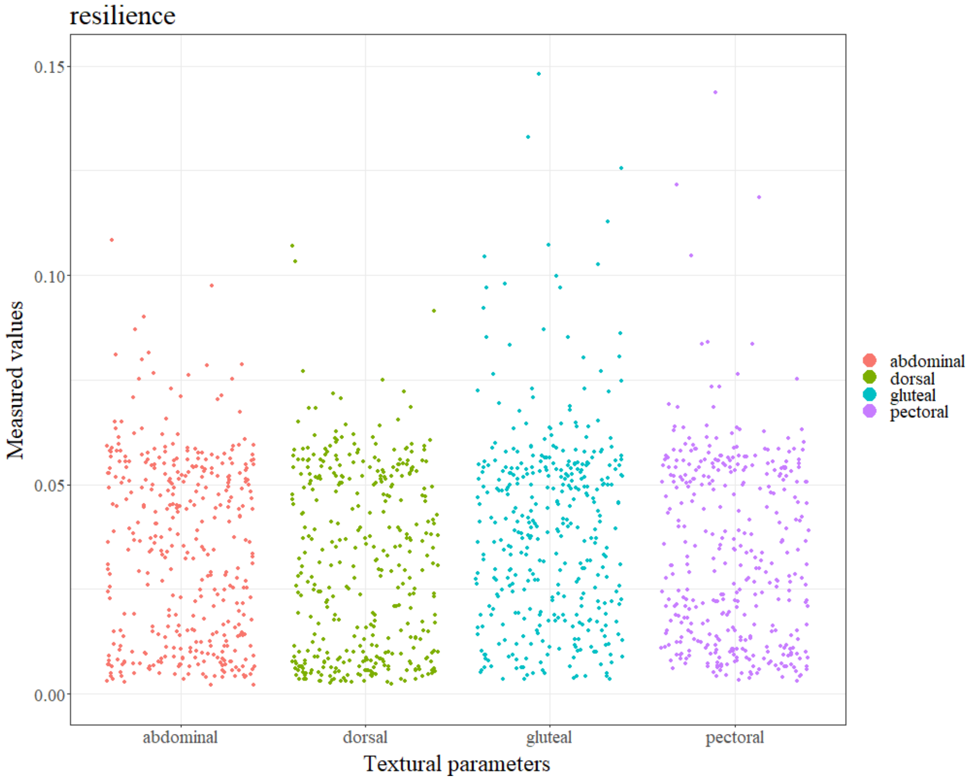

Figure S10. Scatter plot of brittleness values in the four muscle regions of common carp.

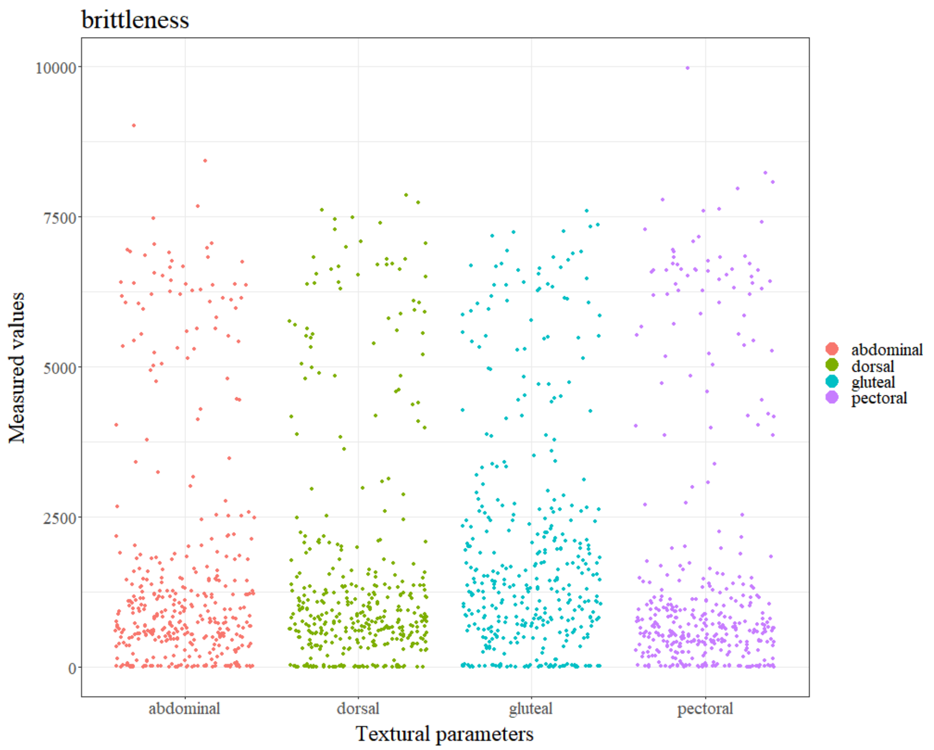

Figure S11. Scatter plot of springiness values in the four muscle regions of common carp.

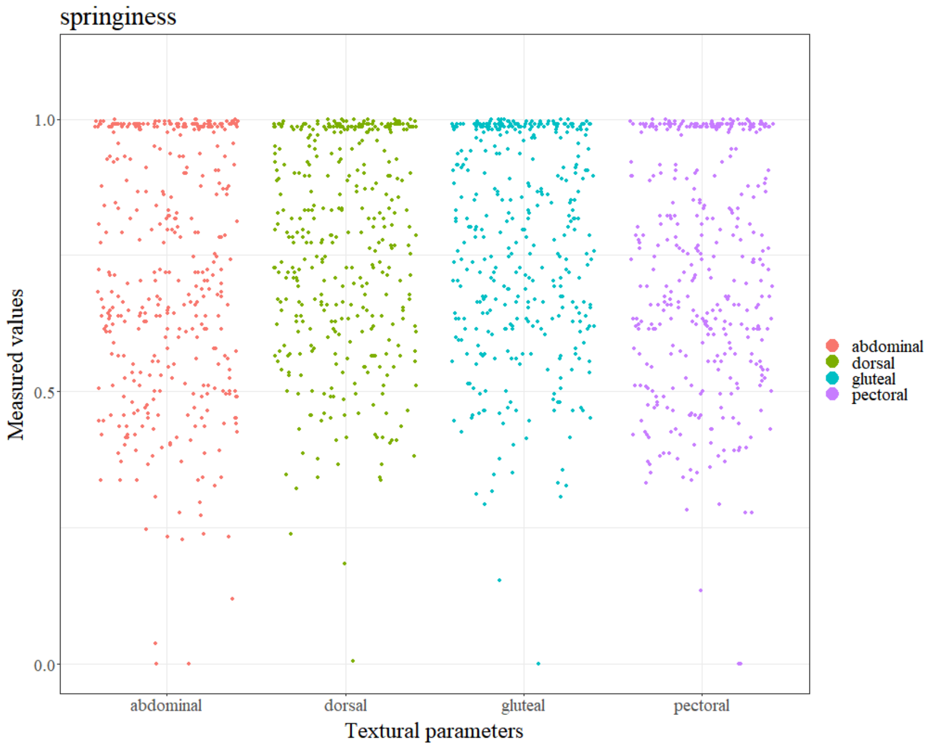

Supplement: Supplementary file 1 [file foods-12-03154-s001.zip › Supplementary Figure.pdf]
