# Supplementary material for: Rapid and Non-Invasive Assessment of Texture Profile Analysis of Common Carp (Cyprinus carpio L.) Using Hyperspectral Imaging and Machine Learning
Source: Foods. 2023 Aug 22;12(17):3154. doi: 10.3390/foods12173154 (PMC10486347; doi:10.3390/foods12173154)
Supplement: Supplementary file 1 [file foods-12-03154-s001.zip › Supplementary Tables.pdf]

Table S3. Correlation analysis of the textural parameters in abdominal muscle of common carp (17 pairs/28).

	gumminess	springiness	cohesiveness	resilience	hardness	brittleness	adhesiveness	chewiness
gumminess		0.013	0.954**	0.027	0.975**	0.580**	-0.862**	0.997**
springiness			0.091	0.728**	-0.053	-0.053	0.031	-0.034
cohesiveness				0.150**	0.909**	0.515**	-0.875**	0.951**
resilience					-0.030	-0.027	0.009	-0.009
hardness						0.614**	-0.843**	0.978**
brittleness							-0.480**	0.574**
adhesiveness								-0.867**
chewiness								

Note: **Correlation is significant at 0.01 levels; *Correlation is significant at 0.05 levels.

Table S4. Correlation analysis of the textural parameters in pectoral muscle of common carp (14 pairs).

	gumminess	springiness	cohesiveness	resilience	hardness	brittleness	adhesiveness	chewiness
gumminess		-0.003	0.080	-0.003	0.37**	-0.030	-0.038	0.097
springiness			0.417**	0.955**	-0.028	-0.052	0.033	-0.036
cohesiveness				0.473**	0.307**	0.492**	-0.771**	0.819**
resilience					-0.022	-0.043	0.024	-0.024
hardness						0.270**	-0.340**	0.385**
brittleness							-0.498**	0.678**
adhesiveness								-0.893**
chewiness								

Table S5. Correlation analysis of the textural parameters in dorsal muscle of common carp (9 pairs).

	gumminess	springiness	cohesiveness	resilience	hardness	brittleness	adhesiveness	chewiness
gumminess		-0.019	0.901**	0.032	0.066	0.002	-0.712**	0.140**
springiness			0.302**	0.921**	-0.004	-0.008	0.032	-0.004
cohesiveness				0.366**	0.080	-0.007	-0.704**	0.120**
resilience					-0.004	-0.008	-0.009	-0.002
hardness						0.001	-0.095	-0.003
brittleness							0.004	-0.002
adhesiveness								-0.127*
chewiness								

Table S6. Correlation analysis of the textural parameters in gluteal muscle of common carp (7 pairs).

	gumminess	springiness	cohesiveness	resilience	hardness	brittleness	adhesiveness	chewiness
gumminess		0.003	0.234**	0.003	0.029	-0.008	-0.020	0.260**
springiness			0.152**	0.990**	-0.014	-0.005	0.003	-0.036
cohesiveness				0.148**	0.120	-0.025	-0.077	0.910**
resilience					-0.015	-0.006	0.004	-0.038
hardness						-0.005	-0.008	0.124*
brittleness							0.000	-0.016
adhesiveness								-0.095
chewiness								
